# Data-Independent Acquisition (DIA) Is Superior for High Precision Phospho-Peptide Quantification in *Magnaporthe oryzae*

**DOI:** 10.3390/jof9010063

**Published:** 2022-12-31

**Authors:** Katharina Bersching, Thomas Michna, Stefan Tenzer, Stefan Jacob

**Affiliations:** 1 Institute of Biotechnology and Drug Research gGmbH (IBWF), Hanns-Dieter-Hüsch-Weg 17, 55131 Mainz, Germany; 2 Institute for Immunology, University Medical Center of the Johannes Gutenberg University Mainz, 55131 Mainz, Germany; 3 Helmholtz-Institute for Translational Oncology Mainz (HI-TRON), 55131 Mainz, Germany; 4 German Cancer Research Center (DKFZ), 69120 Heidelberg, Germany

**Keywords:** proteomics, LC-MS/MS, phospho-peptide enrichment, bioinformatics, cellular signaling, *Magnaporthe oryzae*, phosphorylation, DDA, DIA, phospho-peptidomics

## Abstract

The dynamic interplay of signaling networks in most major cellular processes is characterized by the orchestration of reversible protein phosphorylation. Consequently, analytic methods such as quantitative phospho-peptidomics have been pushed forward from a highly specialized edge-technique to a powerful and versatile platform for comprehensively analyzing the phosphorylation profile of living organisms. Despite enormous progress in instrumentation and bioinformatics, a high number of missing values caused by the experimental procedure remains a major problem, due to either a random phospho-peptide enrichment selectivity or borderline signal intensities, which both cause the exclusion for fragmentation using the commonly applied data dependent acquisition (DDA) mode. Consequently, an incomplete dataset reduces confidence in the subsequent statistical bioinformatic processing. Here, we successfully applied data independent acquisition (DIA) by using the filamentous fungus *Magnaporthe oryzae* as a model organism, and could prove that while maintaining data quality (such as phosphosite and peptide sequence confidence), the data completeness increases dramatically. Since the method presented here reduces the LC-MS/MS analysis from 3 h to 1 h and increases the number of phosphosites identified up to 10-fold in contrast to published studies in *Magnaporthe oryzae*, we provide a refined methodology and a sophisticated resource for investigation of signaling processes in filamentous fungi.

## 1. Introduction

The phosphorylation of proteins is among the most prominent and significant post-translational modifications [1]. Protein kinases make this reversible modification possible by the addition of a phosphate group (PO_4_) to the polar residual of amino acids. As a consequence, this addition modifies the protein from an apolar (hydrophobic) to a more polar (hydrophilic) state. The subsequent conformational changes enable interactions with other molecules [2]. The biochemical nature of phosphorylated amino acids facilitate interaction with other proteins, which enables, e.g., the assembly of proteins or protein complexes. The fundamental challenge in the research of signal transduction pathways is the highly dynamic nature of reversible phosphorylation of the involved signaling proteins [3,4]. Apart from the static information of whether a peptide, peptide fragment or a certain amino acid residue is phosphorylated or not (“on” or “off”), it is of the utmost interest to understand the dynamic alteration of quantitative changes in phosphorylation levels over time associated with a given stimulus or cellular process [5].

In the last decade, liquid chromatography-tandem mass spectrometry (LC-MS/MS) and variations thereof were the method of choice to quantify thousands of proteins across multiple biological samples with high throughput, robustness and sensitivity [6]. An unsolved problem in quantitative phospho-peptidomics by mass spectrometry is still the low abundance of phosphorylated proteins as compared to the complete proteome and to the complement of a given protein, from which naturally only a small portion is phosphorylated in a particular way [7,8,9]. The drive to solve this problem and constantly improve the precision of protein measurements pushes MS-techniques and the related methods forward, following the overarching goal of proteomics to comprehensively identify and quantify all proteins and protein modifications in a biological system [10]. For example, the enrichment of phospho-peptides is absolutely necessary, including immunoprecipitation (IP), metal oxide/immobilized ion affinity chromatography (MOAC/IMAC), fractionation strategies such as high-pH reversed-phase chromatography (HpH RP), strong cation exchange (SCX), or electrostatic repulsion hydrophilic interaction liquid chromatography (ERLIC) [11].

One of the major bottlenecks to obtaining a comprehensive and precise analysis nowadays is not the accuracy of the instruments and measurements used but rather important processes such as data acquisition and data processing [12]. Most of the MS-based proteomic workflows use the “data-dependent acquisition” (DDA) strategy [13,14,15,16], often in combination with “dynamic exclusion” (DE), which rules out a selection of fragmented peptides within a specific time window [17]. In DDA, precursor ions are stochastically selected on the basis of their signal intensity and subsequently fragmented, separated and finally detected by a mass analyzer such as a “time-of-flight” (TOF) or an Orbitrap [18]. In more detail, the top N most intensive *m*/*z* ions are identified from the MS1 scan (precursor spectrum, in proteomics typically precursors are peptides) by the operating software of the mass spectrometer and sequentially selected with a very narrow window (e.g., ±0.5 Dalton) by the quadrupole for fragmentation, so their MS2 spectra (fragment spectra) can be collected. The resulting fragment *m*/*z* values vary by the corresponding masses for amino acids according to their sequence. This way, the processing software (or the analyzing scientist) can compare the obtained amino acid sequence with the measured *m*/*z* of the intact peptide. Depending on the amount of amino acid sequence evidence and the congruence between theoretical and measured precursor *m*/*z*, a score for the probability of a correct identification is calculated [19]. The selected number N of most intense ions is typically between 10 and 25 and can be chosen depending on the instrument speed and on the analytical need. When short LC gradients and highly complex MS1 spectra are present, a high N is needed for deep peptide coverage. On the other hand, a high N costs measurement time and MS1 quantification accuracy. In general, the DDA strategy decides, depending on the MS1 information, which precursors are selected for fragmentation. It provides clean and high-quality spectra that can also be used for de novo sequencing with certain prerequisites. In addition to that, the data processing is not computationally intensive and implements easy and straight forward algorithms that are accessible to a broad community.

In contrast, to alleviate the limitations associated with DDA and DE, strategies on unbiased “data-independent acquisition” (DIA) are available in which every peptide within a specific time window is fragmented [20]. That means that, in data independent acquisition strategy, no preselection is performed. The fragmentation is independent of any MS1 information. Instead of choosing a very narrow window for selecting the precursors for fragmentation, a wide window of precursor *m*/*z* are allowed to pass through the quadrupole [21]. This way, multiple precursors co-fragment and create chimeric MS2 spectra, where the assignment of the precursor and their corresponding fragments is not easily possible. More complex bioinformatic algorithms have to be applied to elucidate the amino acid evidence for each precursor [22]. This also includes the use of spectral libraries, which are either labor intensive or computationally intensive to create. Recent developments in the proteomics community show improvements in algorithms and software to be able to process DIA generated raw data in a comprehensive and user-friendly way. The accessibility of high performing computer systems has paved the way for increasing use of DIA [23]. The major advantage of DIA is a robust and accurate quantification as well as the decrease of missing values, due to the fact that no selection of precursors is performed. Instead, borderline signal intensities are also fragmented and have the chance to be identified and quantified. 

Prior to this study, it was generally assumed that DIA can quantify the same number of proteins as typically identified by DDA methods, but with better accuracy and reproducibility across many samples [24]. In DDA, one major problem was the high number of missing values caused by the experimental procedure due to either a random phospho-peptide enrichment selectivity or borderline signal intensities, which both cause the exclusion for fragmentation. From this follows an incomplete dataset reducing confidence in the subsequent statistical bioinformatic processing.

Here, we successfully developed a method including DIA for data acquisition by using the filamentous fungus *Magnaporthe oryzae* as model organism. Application of this method resulted in an absolutely reliable dataset of *M. oryzae* under osmotic stress with high data quality (such as phosphosite and peptide sequence confidence), while at the same time data completeness increases dramatically. We are convinced that this is an excellent basis for further research on the dynamic processes of phosphorylation in signaling networks in a high quality as never seen before.

## 2. Materials and Methods

### 2.1. Sample Preparation

#### 2.1.1. Cultivation of Magnaporthe Oryzae

The fungal strain used in this study was *Magnaporthe oryzae* (*M. oryzae* 70-15 strain (MoWT), Fungal Genetics Stock Center). The strain was maintained at 26 °C on complete medium (CM) according to [25]. For protein isolation, the *M. oryzae* cultures were grown in 250 mL liquid CM in 500-mL glass flasks for 96 h at 26 °C and 120 rpm. Samples were then taken and the mycelium was immediately separated from the culture fluid and ground into powder with the TissueLyserII (Qiagen) according to the user manual. In order to generate a resource for research on osmotic stress in *M. oryzae*, the samples were stressed by the addition of KCL to a final concentration of 0.5 M, and samples were taken at 0 min (as control sample), 10 min, 60 min, 240 min and 24 h. All samples were generated in biological quadruplicates, making in total 20 samples. In addition to that, three mutated variants with loss of function of MoHOG1, a central osmostress MAPK signaling protein, were included in this research. Details about mutant types and preparation are provided in [26].

#### 2.1.2. Cell Lysis and Protein Digest

If not stated otherwise, all reagents were used in LC-MS/MS grade from common vendors. The sample preparation for all *Magnaporthe oryzae* samples has been performed as described in [11]. In short, a sample aliquot of lyophilized and grinded mycelium was suspended in boiling SDS/DTT lysis buffer with following treatment of ultrasound. Proteins were precipitated by chloroform/methanol precipitation and resolubilized in urea containing buffer. DNA/RNA removal by benzonase and tryptic digest was performed overnight, followed by desalting and lyophilization. An aliquot of lyophilized peptides was used for proteome analysis, and 1000 µg was subjected to phospho-peptide enrichment by TiO_2_ spin tips.

#### 2.1.3. Phospho-Peptide Enrichment

Phospho-peptide enrichment of *M. oryzae* samples was performed as described in [11].

### 2.2. Peptide Identification

#### 2.2.1. LC-MS/MS of M. oryzae Samples for Resource

A total of 3 µL of the reconstituted phospho-peptides were separated on an Ultimate 3000 nanoUPLC (Thermo Scientific, Waltham, MA, USA) with 300 nL/min by a reversed phase C18 column (HSS-T3 C18 1.8 µm, 75 µm × 250 mm, Waters Corporation, Milford, MA, USA) at 55 °C using a 45 min linear gradient from 95% Eluent A (0.1% TFA, 3% DMSO in water) to 35% Eluent B (0.1% TFA, 3% DMSO in ACN) with additional 15 min of equilibration (60 min LC runtime total) followed by ionization in positive mode using a Nanospray Flex electrospray ionization source (Thermo Scientific). Mass-to-charge analysis of the eluting peptides was performed using an Orbitrap Exploris 480 (Thermo Scientific) in data independent acquisition (DIA) mode. MS1 scans were acquired with a resolution of 120,000 at 200 *m*/*z* in a range of 345–1250 *m*/*z*. The RF lens was set to 40% and AGC target to 300% (i.e., corresponding to 3 × 10^6^ charges). DIA MS2 scans were acquired with a resolution of 30,000 at 200 *m*/*z* with a variable window scheme (as shown in supplementary Table S1). The normalized collision energy was set to 27%, RF lens to 40% and AGC target to 1000% (i.e., corresponding to 10 × 10^6^ charges).

#### 2.2.2. LC-MS/MS of M. oryzae DIA Samples for Comparison

A total of 2 µL of the reconstituted phosphor-peptides were separated on a nanoElute LC system (Bruker Corporation, Billerica, MA, USA) at 400 nL/min using a reversed phase C18 column (Aurora UHPLC emitter column, 25 cm × 75 µm 1.6 µm, IonOpticks) which was heated to 50 °C. Peptides were loaded onto the column in direct injection mode at 600 bar. Mobile phase A was 0.1% FA (*v*/*v*) in water and mobile phase B 0.1% FA (*v*/*v*) in can. Peptides were separated, running a linear gradient from 2% to 37% mobile phase B over 45 min. Afterwards, the column was rinsed for 5 min at 95% B followed by equilibration. Eluting peptides were analyzed in positive mode ESI-MS using parallel accumulation serial fragmentation (PASEF) enhanced data-independent acquisition mode (DIA) on a timsTOF Pro 2 mass spectrometer (Bruker Corporation). The dual TIMS (trapped ion mobility spectrometer) was operated at a fixed duty cycle close to 100% using equal accumulation and ramp times of 100 ms each, spanning a mobility range from 1/K_0_ = 0.6 Vs cm^−2^ to 1.6 Vs cm^−2^. We defined 36 × 25 Th isolation windows from *m*/*z* 300 to 1165, resulting in fifteen diaPASEF scans per acquisition cycle. The collision energy was ramped linearly as a function of the mobility from 59 eV at 1/K_0_ = 1.3 Vs cm^−2^ to 20 eV at 1/K_0_ = 0.85 Vs cm^−2^.

#### 2.2.3. LC-MS/MS of M. oryzae DDA Samples for Comparison

A total of 2 μL of the reconstituted phospho-peptides were separated on an Ultimate 3000 nanoUPLC (Thermo Scientific) with 300 nL/min by a reversed phase C18 column (HSS-T3 C18 1.8 μm, 75 μm × 250 mm, Waters Corporation) at 55 °C using a 45 min linear gradient from 95% Eluent A (0.1% TFA/3% DMSO/Water) to 35% Eluent B (0.1% TFA/3% DMSO/ACN), followed by ionization using a Nanospray Flex electrospray ionization source (Thermo Scientific). All samples were measured in triplicates. Mass-to-charge analysis of the eluting peptides was performed using an Orbitrap Exploris 480 (Thermo Scientific) in data dependent acquisition (DDA) mode. Full scan MS1 spectra were collected over a range of 350–1600 *m*/*z* with a mass resolution of 60,000 @ 200 *m*/*z* using an automatic gain control (AGC) target of 100%, maximum injection time was set to “Auto” and RF lens to 40%. Within a fixed cycle time of 1.5 s the most intense peaks above the signal threshold of 2 × 10^4^, harboring a charge of 2–6, were selected within an isolation window of 1.4 Da as precursors for fragmentation using higher energy collisional dissociation (HCD) with normalized collision energy of 30. The resulting fragment ion *m*/*z* ratios were measured as MS2 spectra over an automatically selected *m*/*z* range with a mass resolution of 15,000 @ 200 *m*/*z*, AGC target was set to “Standard” and maximum injection time to “Auto”.

#### 2.2.4. Data Processing Parameters DIA

Peptides were identified and label-free quantification of proteins was performed using DIA-NN (v1.8). Full proteome samples from *M. oryzae* were processed using library free mode with standard parameters, except for tryptic cleavage sites considering no cleavage before proline. The FASTA protein database contained 12.790 protein entries of the *M. oryzae* reference proteome and 172 common contaminant proteins (both from Uniprot). For phospho-peptide analysis of *M. oryzae*, a phospho-peptide spectral library was predicted in silico using the built-in library free prediction algorithm provided by DIA-NN. For *M. oryzae*, the aforementioned FASTA database was used as basis.

The spectra library was predicted with the precursor charge range set between 1 and 4, and the range for fragment ions and precursor mass to charge ratio was limited to 250–1250 *m*/*z*. The peptide length was set to 7–30. Tryptic cleavage considering no cleavage after the lysine or arginine is followed by proline, and maximum one missed cleavage was allowed. N-terminal methionine excision was enabled and cysteine carbamidomethylation was set as fixed modification. The maximum number of variable modifications was set to 3, allowing exclusively UniMod:21 modifications, i.e., mass delta of 79.9663 corresponding to phosphorylation at serine, threonine and tyrosine. The generated spectral libraries were used for follow-up identification and quantification in DIA-NN using the standard settings.

#### 2.2.5. Data Processing Parameters DDA

The DDA raw files were processed by PEAKS X Pro (BSI, Mississauga, ON, Canada) using the FASTA file described in Section 2.2.4. Precursor tolerance and fragment ion tolerance were set to 15 ppm and 0.03 Da, respectively, two missed cleavages were allowed, camabidomethylation at cysteins was set as fixed modification while oxidation on methionine, and phosphorylation on serine, threonine and tyrosine were set as variable modifications with a maximum of 4 variable modifications per peptide.

#### 2.2.6. Availability of Raw Data

All raw data for *M. oryzae* proteome and phosphoproteome resource have been uploaded via JPOST [27] to be available on proteomeXchange [28] and can be accessed with the identifier PXD034481. All files for the DIA/DDA comparison have been uploaded separately to the archive PXD038605.

## 3. Results and Discussion

Comparison of DDA vs. DIA Approach for Phospho-Peptide Identification

**Figure 1:**
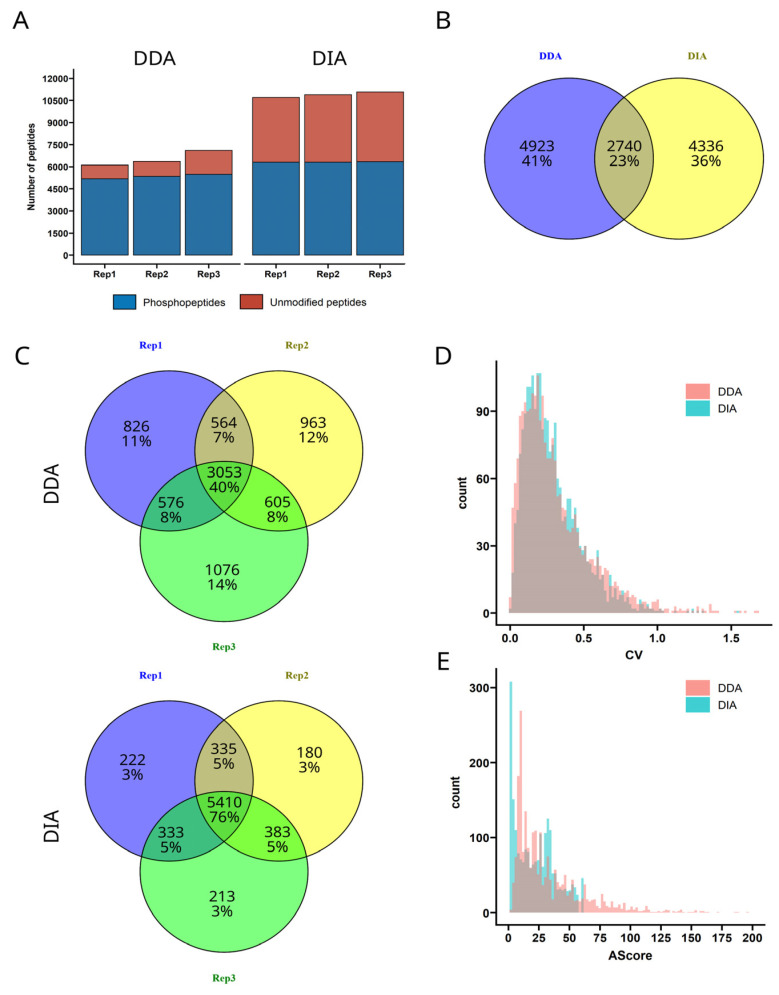
Performance comparison of three *M. oryzae* biological replicates measured in DDA and DIA regarding (**A**) peptide counts (**B**) overlap of identified phosphor-peptides (**C**) overlap within DDA and DIA replicates (**D**) precursor quantity reproducibility and (**E**) phosphosite identification confidence.

A promising approach to gain more confidence in phospho-peptide data is the data independent acquisition (DIA) approach. Per definition, DIA generates MS2 spectra of higher complexity compared to DDA. The identification of the phosphosites especially requires sophisticated bioinformatic methods that had not been available in the past. Recent implementations in proprietary software such as Spectronaut [29], and developments of open source software such as DIA-NN [23], in combination with affordable high-performance computational resources made the analysis of phospho-peptides in DIA possible with sufficient confidence within a reasonable time frame. There are only a few publications describing the use of DIA for phospho-peptides [29,30,31] and thus the differences in the data quality have not yet been reviewed comprehensively, especially in the context of predicted spectral libraries. Furthermore, recent developments in coupling tandem ion mobility spectrometry to high resolution TOF instruments, leading to the commercialization of the timsTOF by Bruker Daltonics, promise a deeper understanding of proteomics datasets by adding an additional identification feature and more confident identification by less complex MS spectra. To investigate the use of DIA for phosphoproteomics in general, and especially the use of the Bruker timsTOF Pro 2, we took the opportunity of available measurement time and produced a dataset of three biological replicates of wildtype *M. oryzae* in DDA with an Orbitrap Exploris 480 and in DIA with a Bruker nanoElute coupled to a timsTOF Pro 2. The datasets were processed with PEAKS and DIA-NN, respectively, and the results summarized in Figure 1.

The number of identified phospho-peptides is comparable between DDA and DIA, while the number of unmodified peptides in the DIA samples is significantly higher. Consequently, the apparent enrichment efficiency decreases from around 80% in DDA to 50% in DIA (Figure 1A). This observation is explained by the DIA scheme, as no criterion for fragmentation is applied, and also unmodified peptides with low signal intensity are selected for fragmentation. Interestingly, all unique phospho-peptide identifications of the three replicates combined are roughly 10% higher in DDA (7.663 peptides) compared to DIA (7076 peptides), and the overlap of peptide IDs is small (23%), as shown in Figure 1B. The overlap of peptide sequences without considering the phosphosite was slightly increased with 44%, so roughly 20% differ in the assigned phosphosite. First, the type of mass spectrometer used certainly influences the selection of peptides to be ionized and thus selected for fragmentation. Additionally, it has not yet been shown to what extent the software DIA-NN actually provides false positive identifications. To exclude a higher false positive rate as the reason for the low number of overlapping identifications, both datasets (DDA and DIA) were searched in either PEAKS or DIA-NN against a connected database of *Mus musculus* and *Magnaporthe oryzae* proteome. As the sample was generated from *M. oryzae*, the number of identified *Mus musculus* proteins are expected to be not more than the previously set up false-discovery rate of 1%. For the DDA dataset, from 36.006 total identifications 112 peptides were identified from *Mus musculus* (i.e., 0.3%) and in the DIA dataset, from 40.817 total identifications only 65 were identified from *Mus musculus* (i.e., 0.2%). In conclusion, the false identification rate can be excluded as a reason for the low overlap between the data acquisition strategies. Comparing the intra-sample group overlap of the identifications within the replicate measurements (Figure 1C) reveals another possible reason for the difference in peptide numbers. DIA consistently provides more reproducible identifications, while the overlap for DDA measurements is much less. When accepting only peptides with at least two out of three identifications, the number of quantifiable peptides is 35% higher in DIA (6.461 peptides) compared to DDA (4.798 peptides), while the number of complete peptide data (three out of three) also increased in DIA measurement. Thus, not only the number of quantifiable peptides but also data completeness is increased.

In order to understand the biology, it is not only the number of quantifiable peptides is important, but also the reproducibility and quality. Therefore, the coefficients of variation (CVs) for every quantifiable peptide (at least two out of three replicates) have been calculated from the replicate measurements and plotted as a histogram (Figure 1D). The difference between both datasets is not significant, with median CVs around 25%, which is reasonable due to technical variability in LC-MS/MS measurement. A beneficial effect of DIA on data quality has been shown on the proteome level [32], which results from the higher number of peptides that are available for quantification. A second important aspect in phospho-peptide identification is the correct localization of the phosphosite. Both approaches, DDA and DIA, offer a confidence measure for the correct site. Nevertheless, even when no evidence for the correct phosphosite is present in the spectrum, the peptide still harbors a phospho-group at some amino acid, otherwise the peptide precursor mass would not be correct. Thus, we can be confident that, due to common quality control measures (e.g., false discovery rate calculation at peptide level), there is a phospho-group present somewhere in the peptide, but the correct phosphosite identification can remain ambiguous. Therefore, DIA-NN calculates a site localization probability and PEAKS provides the Ascore, which is calculated by multiplying the negative decadic logarithm of the *p*-value for incorrect identification by 10. Consequently, the higher the AScore the more confident the identification, with a maximum possible value of 1000. Typically, a confidence of at least 75% (for calculation of AScore: 25% probability of false localization) is desired [33] as a prerequisite for class I phosphosite. Therefore, a common cut of value for the AScore is a value of 6, corresponding to 25% false localization probability. In Figure 1E, the distribution of AScores obtained from both acquisition strategies is shown. The DDA AScores peak is around a value of 10, whereas DIA data seem to provide two different peaks, the first peak with an AScore below 6 and the second peak with an AScore around 30, which equals a site confidence of 99.9%. Thus, the median site confidence is roughly the same, due to the inhomogeneous distribution of the DIA-NN confidences. The reason for this difference is presumably the higher complexity of MS2 in DIA data. There, confidence is only achieved in the presence of strong fragment evidence, whereas the algorithm of PEAKS for processing DDA MS2 spectra seems to have a more refined algorithm to assign calculate variances in probability with high sensitivity. A possible reason for the clear separation of either very low confidence or very high confidence of the phosphosite localization in DIA analysis with ion mobility included might be a combination of ion mobility separation before fragmentation, yielding in cleaner spectra, and the de-noising capability of MS2 spectra in DIA-NN, that contributes to increased identification of evidence fragments for the correct phosphosite. Therefore, the assumption that DDA data provide more confidence in the site localization by higher quality spectra is only partly true. Nevertheless, discovery phase in phospho-proteomics, the correct phosphorylation site is of less importance anyway. More importantly, both algorithms provide equally high confidence that these peptides are phosphorylated (regardless of the phosphosite). Conclusions about active/inactive pathways or protein phosphorylation with approximate protein sites can be drawn anyway.

Based on these findings, we measured a sample set of *M. oryzae* samples including KCL salt stress to build a resource for further research. Across all samples, 29.494 unique phospho-peptides could be identified, corresponding to a total number of 45.291 phosphosites. The most recent phosphoproteomics study in *M. oryzae* from 2015 by W.L. Franck et al. in the group of R.A. Dean [34] reported 4894 phosphosites, which we were able to increase, outperforming by a factor of roughly 10-fold with our methodology. In addition to that, W.L. Franck et al. used a chromatography method which took as long as 3 h, which we could outperform by a factor of 3-fold by developing an LC method with only a 45 min gradient and 60 min runtime in total.

In conclusion, the application of DIA is a promising strategy for the comprehensive description of a phospho-proteomics dataset. We have shown that data completeness increases dramatically while the data quality remains at least equal. The downsides of the DIA application are resource intensive and time consuming bioinformatic processing and the lack of intuitive spectra visualization. A possible solution to this is provided by the proprietary software Spectronaut, which is able to visualize XICs of precursors and fragments in a user-friendly way [29]. Nevertheless, DIA-NN has been shown to provide superior identification performance, utilizing neuronal networks while being open source at the same time. A direct phospho-peptide ID benchmark of both types of software has not been described in the literature yet and would serve as interesting starting point for further bioinformatics research.

Furthermore, we not only provide a refined methodology for phospho-peptide analysis in filamentous fungi but also a large dataset that can serve as valuable resource for further signaling research in *M. oryzae*.

## Data Availability

All raw data have been uploaded via JPOST [27] to be available on proteo-meXchange [28] and can be accessed with the identifier PXD034481.

## Supplementary Materials

The following supporting information can be downloaded at: https://www.mdpi.com/article/10.3390/jof9010063/s1, Table S1: Variable window sizes foe DIA acquisition with Orbitrap Exploris 480.
