# Supplementary material for: Data-Independent Acquisition (DIA) Is Superior for High Precision Phospho-Peptide Quantification in Magnaporthe oryzae
Source: J Fungi (Basel). 2022 Dec 31;9(1):63. doi: 10.3390/jof9010063 (PMC9863866; doi:10.3390/jof9010063)
Supplement: Supplementary file 1 [file jof-09-00063-s001.zip › jof-2051724-supplementary.pdf]

Table S1: Variable window sizes foe DIA acquisition with Orbitrap Exploris 480.

Window	center (m/z)	Isolation Window (m/z)	m/z start	m/z end
1	392.58	95.2	344.98	440.18
2	459.56	39.8	439.66	479.46
3	494.25	30.6	478.95	509.55
4	522.78	27.5	509.03	536.53
5	548.53	25	536.03	561.03
6	572.89	24.7	560.54	585.24
7	596.9	24.3	584.75	609.05
8	620.81	24.5	608.56	633.06
9	645.09	25.1	632.54	657.64
10	670.1	26	657.1	683.1
11	696.1	27	682.6	709.6
12	723.35	28.5	709.1	737.6
13	752.37	30.6	737.07	767.67
14	783.12	31.9	767.17	799.07
15	816.29	35.4	798.59	833.99
16	852.84	38.7	833.49	872.19
17	893.43	43.5	871.68	915.18
18	939.95	50.6	914.65	965.25
19	997.57	65.7	964.72	1030.42
20	1070.86	81.9	1029.91	1111.81
21	1180.66	138.7	1111.31	1250.01
